# Comparative Transcriptome Analysis in the Hepatopancreas Tissue of Pacific White Shrimp *Litopenaeus vannamei* Fed Different Lipid Sources at Low Salinity

**DOI:** 10.1371/journal.pone.0144889

**Published:** 2015-12-15

**Authors:** Ke Chen, Erchao Li, Zhixin Xu, Tongyu Li, Chang Xu, Jian G. Qin, Liqiao Chen

**Affiliations:** 1 Laboratory of Aquaculture Nutrition and Environmental Health, School of Life Sciences, East China Normal University, Shanghai 200241, China; 2 School of Biological Sciences, Flinders University, Adelaide, SA 5001, Australia; Chinese Academy of Fishery Sciences, CHINA

## Abstract

RNA-seq was used to compare the transcriptomic response of hepatopancreas in juvenile *Litopenaeus vannamei* fed three diets with different lipid sources, including beef tallow (BT), fish oil (FO), and an equal combination of soybean oil + BT + linseed oil (SBL) for 8 weeks at 3 practical salinity unit (psu). A total of 9622 isogenes were annotated in 316 KEGG pathways and 39, 42 and 32 pathways significantly changed in the paired comparisons of FO vs SBL, BT vs SBL, or FO vs BT, respectively. The pathways of glycerolipid metabolism, linoleic acid metabolism, arachidonic acid metabolism, glycerophospholipid metabolism, fatty acid biosynthesis, fatty acid elongation, fatty acid degradation, and biosynthesis of unsaturated fatty acid were significantly changed in all paired comparisons between dietary lipid sources, and the pathways of glycerolipid metabolism, linoleic acid metabolism, arachidonic acid metabolism and glycerophospholipid metabolism significantly changed in the FO vs SBL and BT vs SBL comparisons. These pathways are associated with energy metabolism and cell membrane structure. The results indicate that lipids sources affect the adaptation of *L*. *vannamei* to low salinity by providing extra energy or specific fatty acids to change gill membrane structure and control iron balance. The results of this study lay a foundation for further understanding lipid or fatty acid metabolism in *L*. *vannamei* at low salinity.

## Introduction

As a euryhaline penaeid species, the Pacific white shrimp *Litopenaeus vannamei* has become an emerging species for crustacean farming in inland low salinity water [1]. Although relatively high production of *L*. *vannamei* has been achieved, economic profit is hindered by slow growth and low survival [2], low immune ability [3], and low stress resistance [2, 4] at low salinity. Various studies have been conducted on *L*. *vannamei* growth and survival [5–7], immune response [8, 9] and nutritional requirements at low salinity [10–15], but little is known on the mechanism of physiological adaptation to the change of dietary nutrients from the perspective of metabolism pathways.

Among dietary nutrients, lipids are of the highest energy, and contain various essential fatty acids for growth and development in aquatic animals. The content of arachidonic acid (20:4n-6; AA) in gills is important for osmoregulation [16], and docosahexaenoic acid (22:6n-3; DHA) and eicosapentaenoic acid (20:5n-3; EPA) can increase the gill area and enzymatic efficiency to improve osmoregulation [17, 18]. Among lipid types, phospholipid and glycolipid are the indispensable components for cell membrane structure, and lipid composition affects osmoregulation capacity [19]. Therefore, lipids are functionally important in response to ambient salinity shock in aquatic animals, especially at low salinity [20]. Physiological functions of lipids are closely related to the relative components of fatty acids, triacylglycerol, phospholipid, cholesterol and phosphoglycerides in different tissues. The impact of lipid sources on *L*. *vannamei* growth performance has been evaluated at different salinities based on weight gain, feed conversion, survival and fatty acid composition [21, 22]. However, the optimal source of lipids for *L*. *vannamei* and the physiological response to different lipids at the salinity less than 5 psu are poorly known.

In contrast to freshwater species, marine species have a limited ability to synthesize long chain (>20 carbons) polyunsaturated fatty acids (LC-PUFAs) [23], and EPA and DHA are essential to marine animals [24]. Therefore, salinity may functionally important in regulating the synthesis of long chain PUFAs [25]. In a marine teleost, *Siganus canaliculatus* can convert C18 PUFA to LC-PUFA, and this activity is enhanced by decreasing salinity from 32 to 10 psu [25]. The desaturase and elongase enzymes required for synthesizing DHA from C18 PUFA in *S*. *canaliculatus* have been identified [26, 27], including Δ4 fatty acyl desaturase (Δ4 Fad) and a bifunctional Δ6/Δ5 Fad [27]. In *L*. *vannamei*, both linolenic (C18:3n-3) in hepatopancreas and EPA (C20:5n-3) in muscle at 3 psu were significantly higher than those at 30 psu [20]. However, the potential ability of *L*. *vannamei* to synthesize LC-PUFA (>20 carbons) from C18 PUFA has not been revealed and the impact of ambient salinity on carbon chain elongation in shrimp is not clearly.

As a practical and efficient method to obtain the relatively complete genes and complex molecular pathways involved in physiological function [28–30], RNA sequencing (RNA-seq) has been applied in various aquatic animals [31–34], including *L*. *vannamei* [35], taura syndrome virus [36], and white spot syndrome virus [37]. In a previous study in our lab, the *L*. *vannamei* fed a diet with an equal combination of soybean oil +BT+ linseed oil (SBL) as the lipid source showed the highest weigh gain compared with other single lipid sources[38], but the molecular mechanism remains unknown. This study was a continuation of the previous study to understand the transcriptome response of hepatopancreas in *L*. *vannamei* to the source of dietary lipids. This is the first attempt to use transcriptome analysis to reveal the key pathways and genes sensitive to the change of dietary lipid sources in *L*. *vannamei* at low salinity. The results would lay a useful foundation to further understand the lipid or fatty acid metabolism in *L*. *vannamei* at low salinity.

## Materials and Methods

### Experimental animals, design and facilities

Juvenile white shrimp (1.86 ± 0.32 g) were obtained from the Shenzhen base of South China Sea Fisheries Research Institute, Shenzhen, China, and were stocked in nine tanks at a density of 40 shrimps per tank (500 L) at 17 psu salinity for one week. Then shrimp were acclimated to 3 psu by a daily change of 2 psu prior to the start of the 8-week experiment. During the acclimation period, shrimp were fed three times daily at 0800, 1600 and 2200 h with a commercial diet (10% moisture, 40% crude protein, 8% crude lipid, 12% ash, 30% carbohydrates, 16.7 kJ g^-1^ digestible energy), and when the experimental period started, shrimp were fed three times daily at 0800, 1600 and 2200 h with three purified diets containing different fatty acid contents (S1 and S2 Tables). Based on the amount of uneaten feed on the previous day, the daily ration was adjusted to a feeding level slight over satiation. The unfed feed was daily removed with a siphon tube. The photoperiod was 12 h light and 12 h dark. Seawater was pumped from the Daya Coast in Shenzhen and filtered through an activated carbon cartridge for at least 3 d before entering the culture system. Tap water was aerated before it was added to the tank to adjust the salinity level. During the experiment, water was exchanged once daily with 1/3 of the tank volume. Water quality parameters were monitored 2–3 times a week throughout the feeding trial, and maintained at pH 7.5–7.9, temperature 26–28°C, dissolved oxygen 4.8–6.4 mg/L, and total ammonia nitrogen <0.02 mg/L during the trial.

At the end of the experiment, shrimp were deprived of feed for 24 h before sampling. Five shrimp at intermolt stage C in each tank were dissected to obtain the hepatopancreas for transcriptome analysis. The hepatopancreas were carefully taken out from the shrimps by a sterilized tweezer and encased the hepatopancreas into a sterilized EP tube, then put the EP tube in the liquid nitrogen and stored at -80°C for RNA extraction.

### RNA extraction, library preparation and Illumina Hiseq2500 sequencing

Total RNA was extracted from the tissue of hepatopancreas by using the TRIzol® reagent according the manufacturer’s instructions (Invitrogen) and genomic DNA was removed using DNase I (TaKara). Then RNA quality was determined by 2100 Bioanalyser (Agilent) and quantified using the NanoDrop 2000 (ND-2000, Gene Company limited). Only the high-quality RNA sample (OD260/280 = 1.8~2.2, OD260/230 ≥2.0, RIN ≥6.5, 28S:18S ≥1.0, >10 μg) was used to construct the sequencing library.

RNA-seq transcriptome library was prepared following the TruSeq^TM^ RNA sample preparation instruction from Illumina (San Diego, CA) using 5 μg of total RNA. Shortly, messenger RNA was isolated according to the polyA selection method by oligo (dT) beads and then firstly segmented (100 to 400 bp) by a fragmentation buffer. Secondly double-stranded cDNA was synthesized using a SuperScript double-stranded cDNA synthesis kit (Invitrogen, CA) with random hexamer primers (Illumina). Then the synthesized cDNA was subject to end-repair, phosphorylation and ‘A’ base addition according to Illumina’s library construction protocol. Libraries were size-selected for cDNA target fragments of 200–300 bp on 2% low range ultra-agarose followed by PCR amplification using Phusion DNA polymerase (NEB) for 15 PCR cycles. After being quantified by TBS380, the paired-end RNA-seq library was sequenced with the Illumina HiSeq 2500 (2 × 100 bp read length). Raw reads were archived at the National Center for Biotechnology Information’s Sequence Read Archive under the accession No. SRP048814.

### De novo assembly and annotation

The raw paired end reads were trimmed and quality controlled by SeqPrep (https://github.com/jstjohn/SeqPrep) and Sickle (https://github.com/najoshi/sickle) with default parameters. Then clean data from the samples were used to do RNA de novo assembly with Trinity (http://trinityrnaseq.sourceforge.net/) [39]. All the assembled transcripts were searched against the NCBI protein nonredundant (NR), String and KEGG databases using BLASTX to identify the proteins that had the highest sequence similarity with the given transcripts to retrieve their function annotations. A typical cut-off E-value was set at <1.0×10^−5^. The BLAST2GO (http://www.blast2go.com/b2ghome) [40] program was used to obtain GO annotations of unique assembled transcripts for describing biological processes, molecular functions and cellular components. Metabolic pathway analysis was performed using the Kyoto encyclopedia of genes and genomes (KEGG, http://www.genome.jp/kegg/) [41].

### Differential expression analysis and functional enrichment

To identify differential expression genes (DEGs) between two samples, the expression level of each transcript was calculated according to the fragments per kilobase of exon per million mapped reads (FRKM) method. RSEM (http://deweylab.biostat.wisc.edu/rsem/) [42] was used to quantify gene and isoform abundances. R statistical package software EdgeR (empirical analysis of digital gene expression in R, http://www.bioconductor.org/packages/2.12/bioc/html/edgeR.html) [43] was used for differential expression analysis. In addition, functional-enrichment analysis including GO and KEGG was performed to identify which DEGs were significantly enriched in GO terms and metabolic pathways at Bonferroni-corrected P-value ≤ 0.05 compared with the whole-transcriptome background. GO functional enrichment and KEGG pathway analysis were carried out by Goatools (https://github.com/tanghaibao/Goatools) and KOBAS (http://kobas.cbi.pku.edu.cn/home.do) [44].

### Experimental validation of RNA-seq profiles by qPCR

Fifteen randomly selected genes with significant expression from the KEGG pathways were used for validation by real-time qPCR. The gene-specific primers were designed by Primer Premier 6 (Table 1). Total RNA was extracted from the target hepatopancreas tissues using a TRIpure Reagent kit (Aidlab, RN01). Samples of polyadenylated RNA were reverse-transcribed using a TaKaRa kit (Cat. No. RR036A). Reactions were carried out in a total volume of 20 μl, and the volumes of the reaction components were as follows: 2 μl 5X PrimeScript RT Master Mix (Perfect Real Time), 1 μg total RNA, and followed by adding RNase-free dH_2_O up to 20 μl. The protocol for reverse transcription was 37°C for 15 min, 85°C for 5 s, and 4°C for the rest of time. The qPCR was carried out in the CFX96^TM^ Real-Time PCR system (Bio-Rad Laboratories, Richmond, CA) using Ultra SYBR Mixture (WCBIO, CW0957). The amplifications were performed in a 96-well plate in a 20 μl reaction volume containing 10 μl of UltraSYBR Mixture (WCBIO, CW0957), 0.4 μl (each) gene-specific forward and reverse primers, 8.4 μl RNase-free water and 0.8 μl cDNA. The thermal profile for UltraSYBR Mixture PCR was 95°C for 10 min followed by 40 cycles of 95°C for 15 s and 60°C for 1 min. The β-Actin gene was used as the reference gene and each gene had three replicated wells. Relative fold changes were calculated in the Relative Expression Software Tool version 2009 based on the cycle threshold values generated by qPCR [45].

**Table 1:** Primers used for qPCR analysis.

Gene name	Sense Primer	Anti-sense Primer	Product size
CYP3A	CTTGCTGTCCAGTGTGGTCCTA	GTTGGTGGTTGCTGCCGTATAG	128
ACAD8	GCCAGGTTCAGGATCAGATGCT	CACCACCTCCGCTTATGAATGC	104
FLT1	CCTCCTACAACCACCAGCAGAT	GCCATCCTTGAACCACACGAAC	92
BHMT	TTCGTGTTCGCCCTGGAGAA	AGAAGGTGAACGCTTGCATGAC	145
MLL2	CGTGAAGATGTGGCTGGAGATG	TAGACTAGGCTGGCGAGGACTT	138
XDH	CTCAGCATTGACGAGTCCGAAG	TGACCCACGCAGGTAACTTTCT	149
E1.14.11.1	TGACATCCACCGACGCCTATT	GGCAGACTTCCTTGTTGCTGTT	123
SLC17A5	TGGCGTGAGGTGTTCCTGAT	TTGTCATCGGCGTTGCTCTG	134
AP1G1	GGCATACTTGTTGGACGGTCTG	AGGTGTTGTGAGCGTGTTGGA	97
SMPD1	AAGATTGAGACGCCCAGAGTGT	TGCCACAGATGTCACCGATGA	130
CYP2J	AGACCTACCTGGAGGAGAGCAT	TGCGACCAACTGCCAGATGA	137
CYP3A	ACTCCTTCCACGAGCCATTGTT	CGTCCTTCTTGTTCGGCATGTT	120
DOT1L	TGGCAAGCAGCACAGTGAGTA	AGGCGAAGTTGTTGACGAAGAC	107
MLL3	ACGAAGAGGAGGACGAGGAGAA	GGCTCAGGACCAGGCAATGTAT	148
CTSC	AGCAACCACCAGAAGCCAGTT	GTTCTCCAGCACAACACCAACA	135

## Results

### Sequencing and de novo assembly and validation of RNA-seq results by qPCR

A total of 140.18 million reads were obtained from the hepatopancreas of *L*. *vannamei* (Table 2). After quality trimming and adapter clipping, a total of 135.51 million high quality reads remained. In total, 26,034 genes and 38,237 isogenes with the average length of 1,610 bp were obtained after splicing and removing redundancy (Table 3). The length distribution of isogenes is shown in S1 Fig. The mapping data of the assembly isogenes were over 90% of the total, showing that the transcriptome data set had commendable gene coverage.

**Table 2:** Summary of Illumina expressed short read production and filtering of transcriptomic responses to low salinity stress in Litopenaeus vannamei.

Salinity	Reads	nucleotides	Q20 (%)	Q30 (%)
BT	47,256,862	4,772,943,062	96.57	91.34
FO	45,745,774	4,620,323,174	96.81	91.79
SBL	47,190,680	4,766,258,680	96.87	92.02
Trimmed				
BT	45,620,924	4,501,250,936	99.09	94.57
FO	44,250,222	4,372,768,553	99.13	94.80
SBL	45,643,340	4,513,906,418	99.15	94.96

Note: Q20 means that every 100 bp sequencing reads will have an error and Q30 means that every 1000 bp sequencing reads will have an error.

**Table 3:** Summary of de novo assembly results of transcriptomic responses to salinity stress in Litopenaeus vannamei.

Type	Number
Total genes:	26034
Total isogenes:	38237
Total residues:	61573030 bp
Average length:	1610.3bp
Largest isogene:	24554bp
Smallest isogene:	351bp

**Fig 1:**
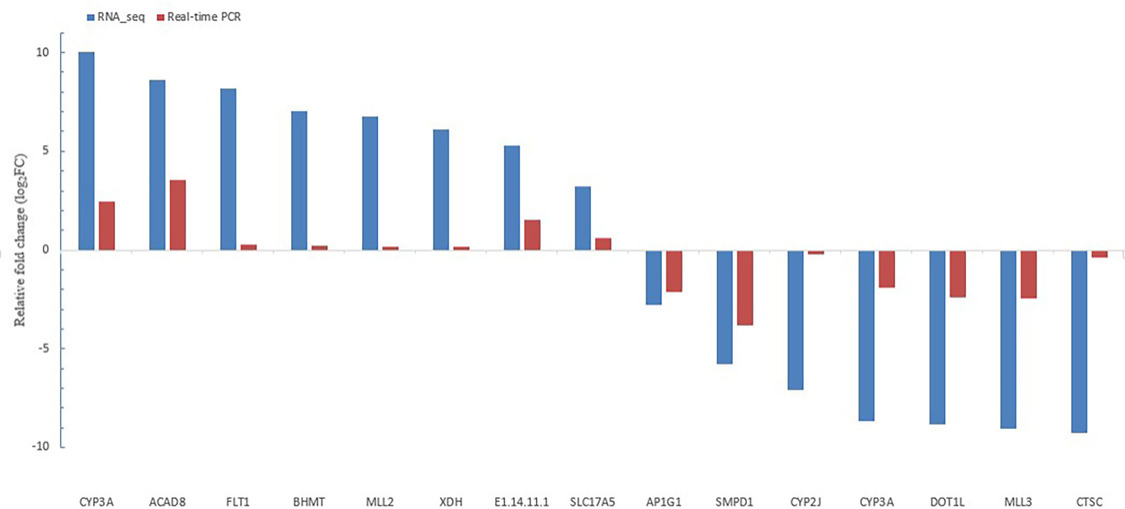
Validation results of RNA-seq profiles by qPCR.

Fifteen randomly selected genes were determined with same hepatopancreas RNA samples by qPCR. All these genes were significantly associated with the RNA-seq results (*R* = 0.77, Fig 1). These results also further confirmed the reliability of RNA-seq and the accuracy in Trinity assembly.

### Annotation of isogenes

Among the annotated and predictable sequences, a total of 17,232 (76.83%), 6,298 (28.08%), 3,720 (16.58%) and 302 (1.35%) sequences were unambiguous alignments relative to the reference after BLASTx against NR and string, KOG, COG and NOG databases, respectively (Table 4). However, among unpredictable sequences, only 2,235 (14.14%), 746 (4.72%), 509 (3.22%), 313 (1.98%), 243 (1.54%), 128 (0.81%), 56 (0.35%) of the total 15,806 sequences were matched against NR, GO, NT, string, KOG, COG and NOG databases, respectively (Table 4).

**Table 4:** Summary of the annotations of Litopenaeus vannamei isogenes.

	Predicted sequences		Unpredictable sequences	
	Number	Ratio (%)	Number	Ratio (%)
All genes	22431	100	15806	100
Annotated in NR	17232	76.82	2235	14.14
Annotated in NT	None	None	509	3.22
Annotated in GO	None	None	746	4.72
Annotated in string	6298	28.08	313	1.98
Annotated in COG	3720	16.58	128	0.81
Annotated in KOG	5408	24.11	243	1.54
Annotated in NOG	302	1.35	56	0.35

Analysis of COG annotation showed that three types of function were obtained including information storage and processing, cellular processes and signaling, and metabolic pathways. The hits from COG prediction were functionally classified into 25 categories, in which most enriched terms were in general functions, followed by transcription and signal transduction mechanisms (S2 Fig).

### KEGG pathway annotation and functional enrichment analysis

**Fig 2:**
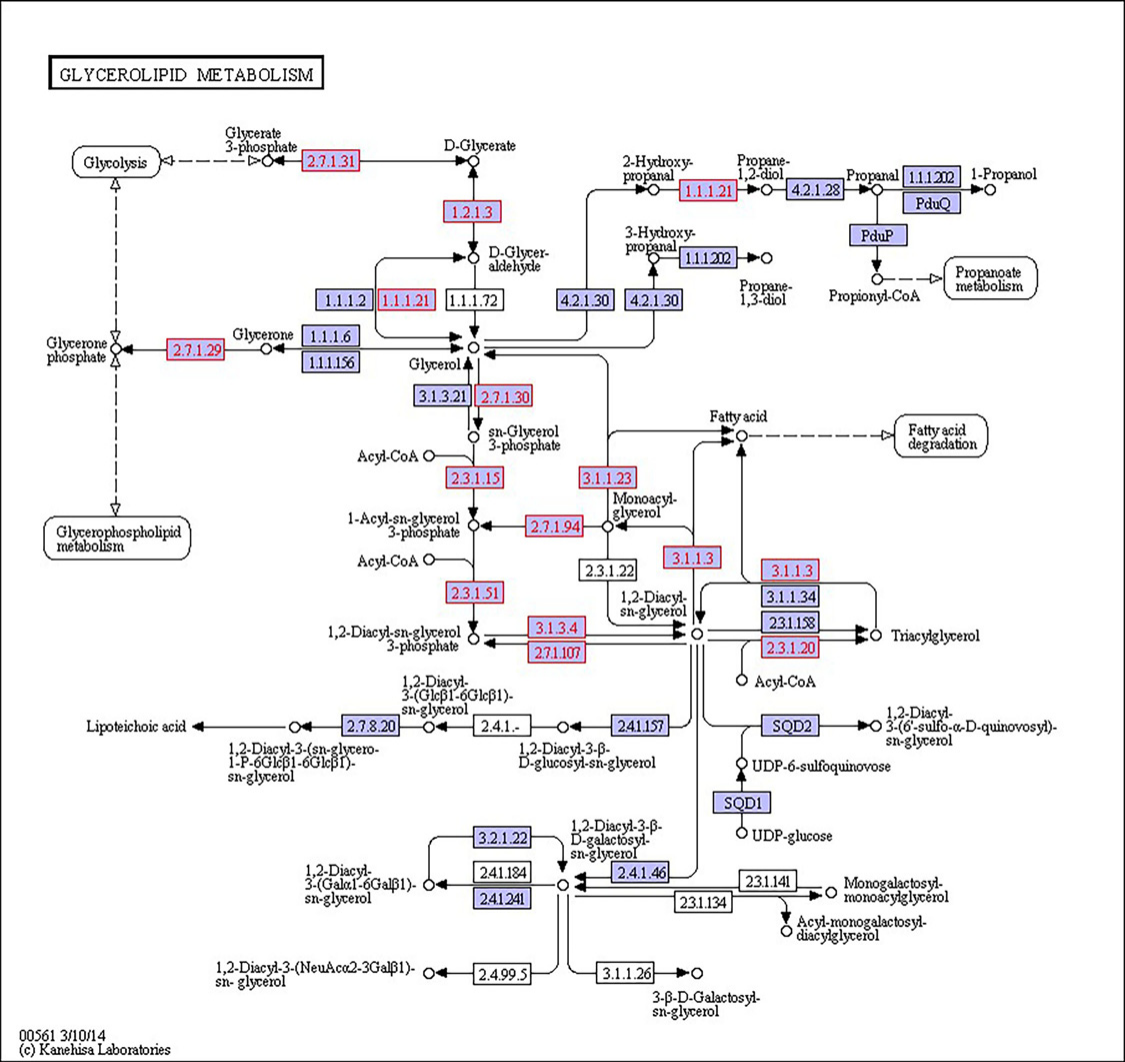
Pathway of glycerolipid metabolism.

**Fig 3:**
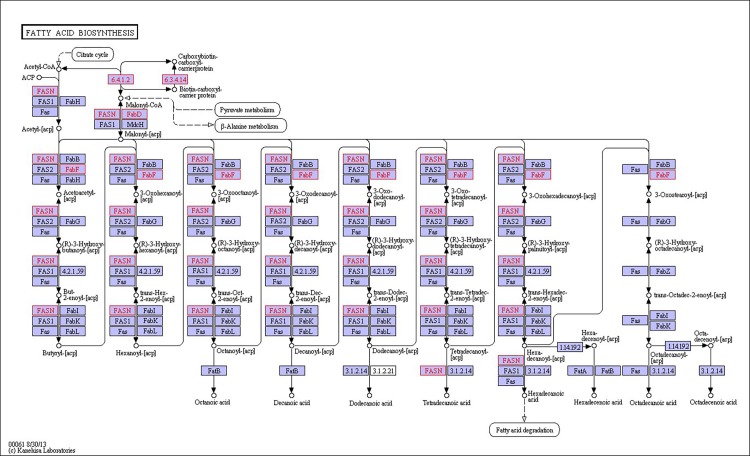
Pathway of fatty acid biosynthesis.

**Fig 4:**
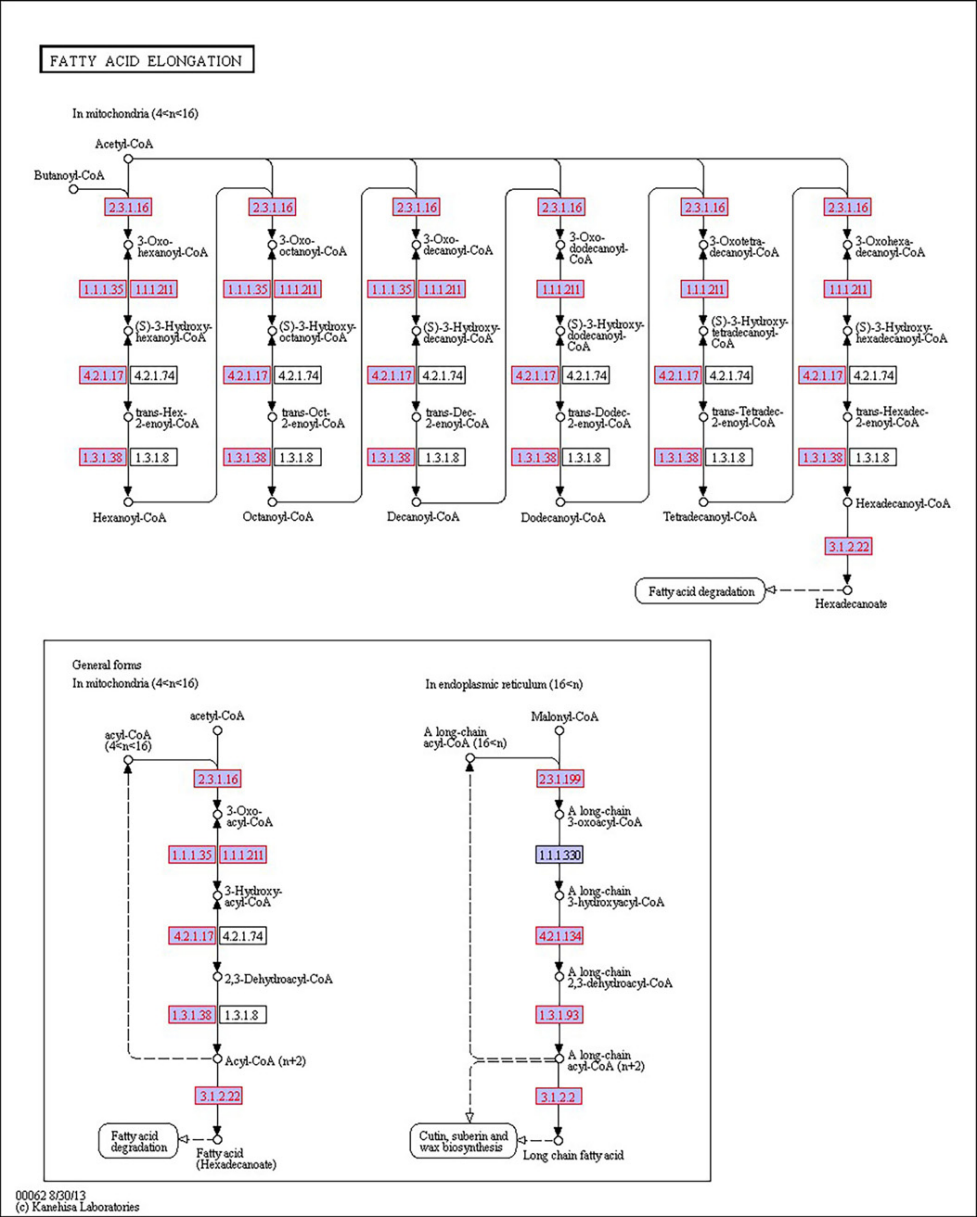
Pathway of fatty acid elongation.

**Fig 5:**
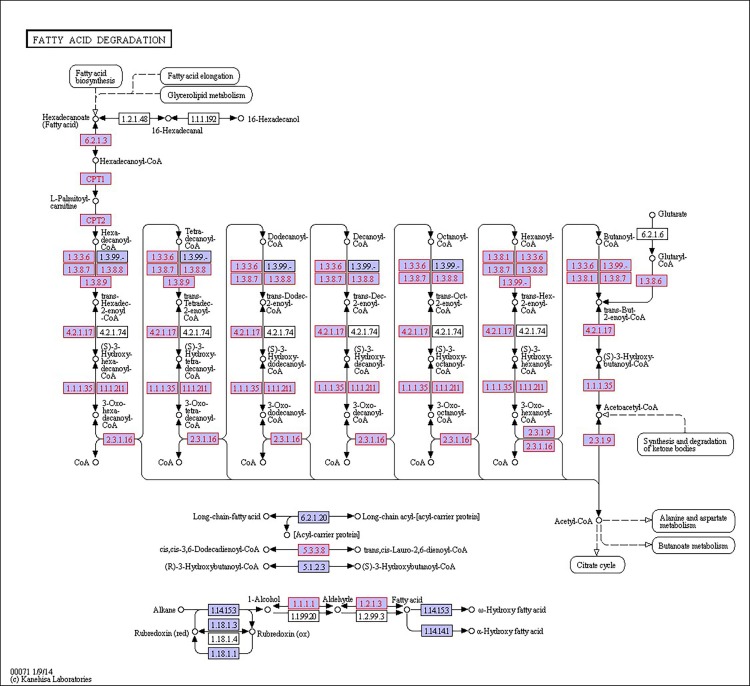
Pathway of fatty acid degradation.

**Fig 6:**
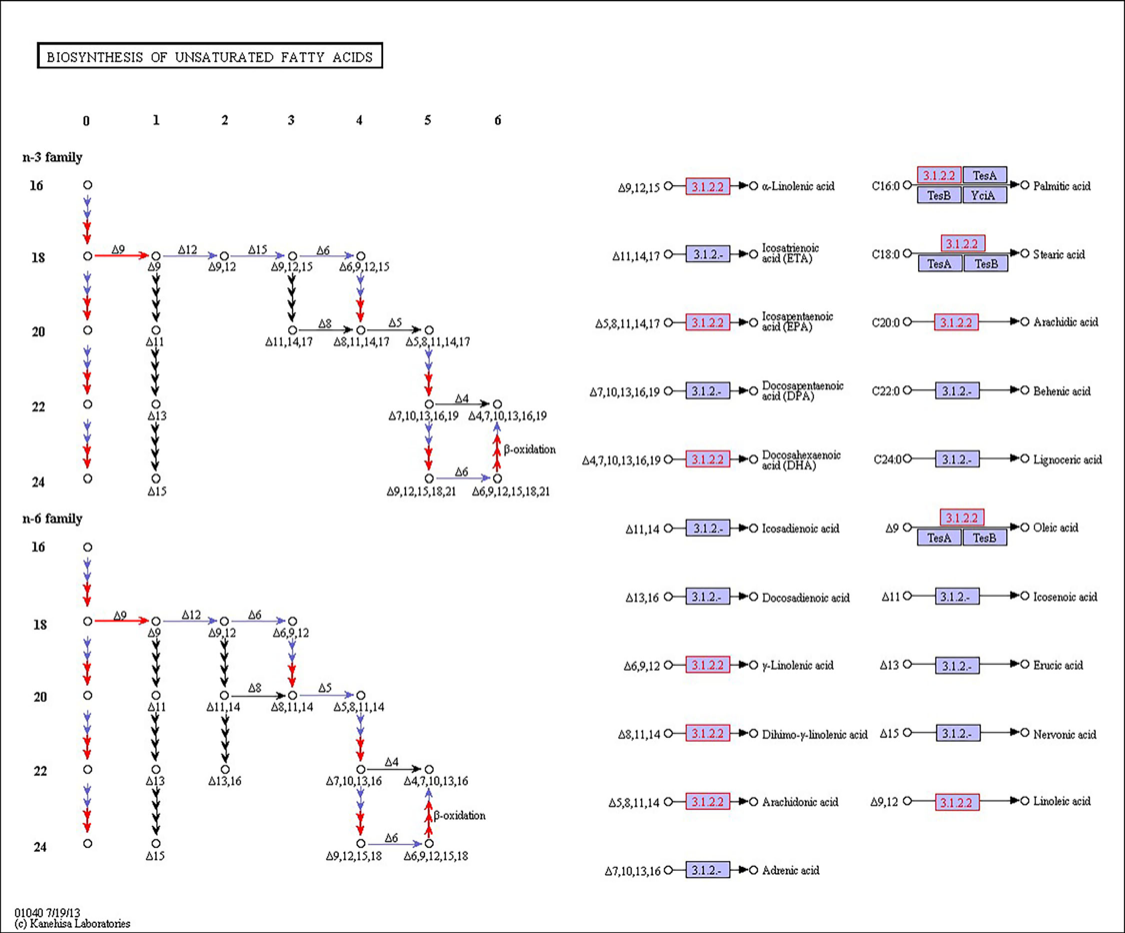
Pathway of biosynthesis of unsaturated fatty acid.

**Fig 7:**
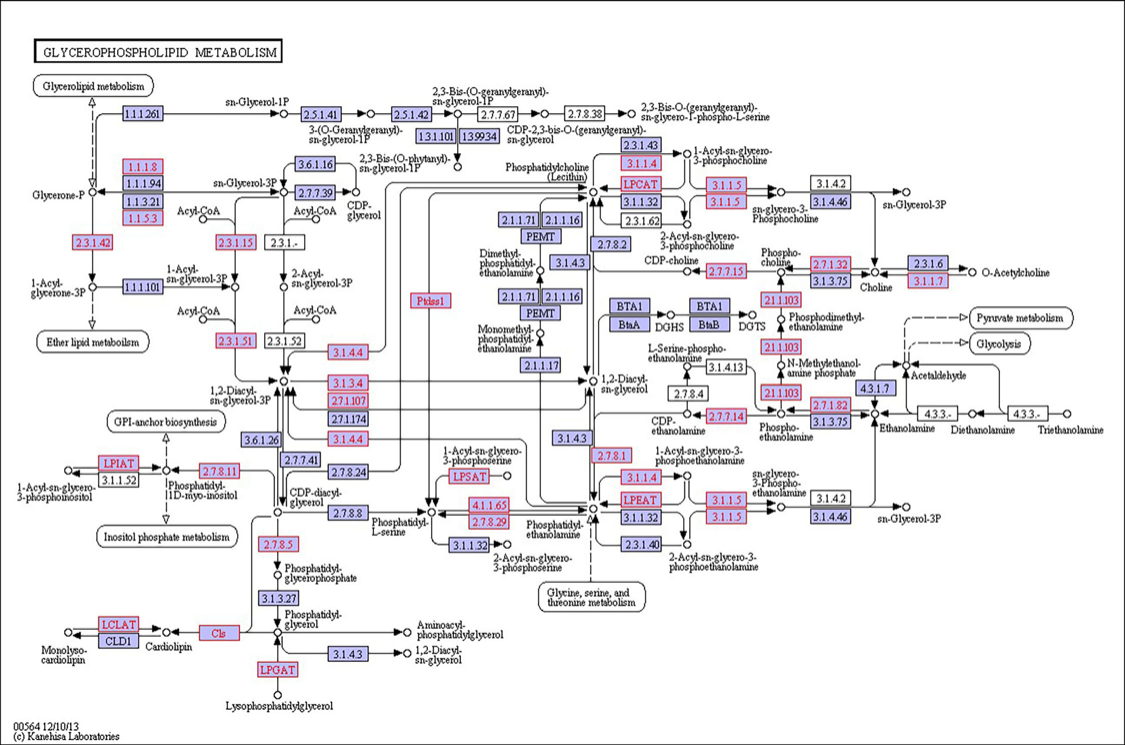
Pathway of glycerophospholipid metabolism.

**Fig 8:**
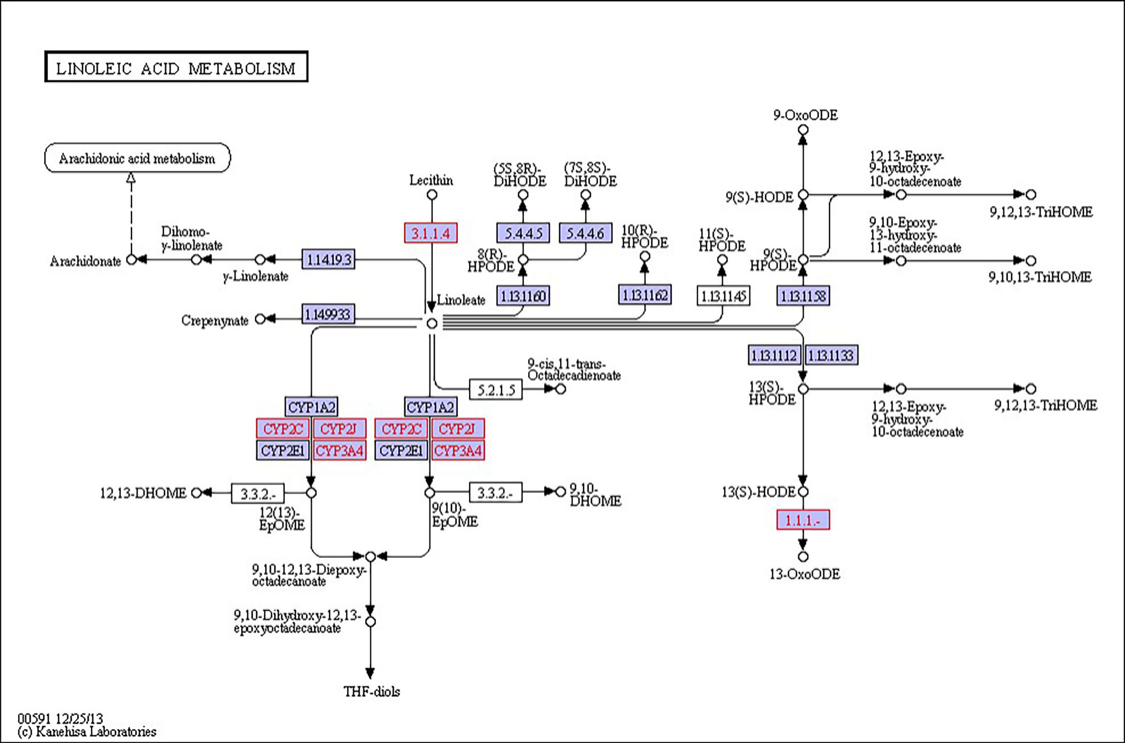
Pathway of linoleic acid metabolism.

**Fig 9:**
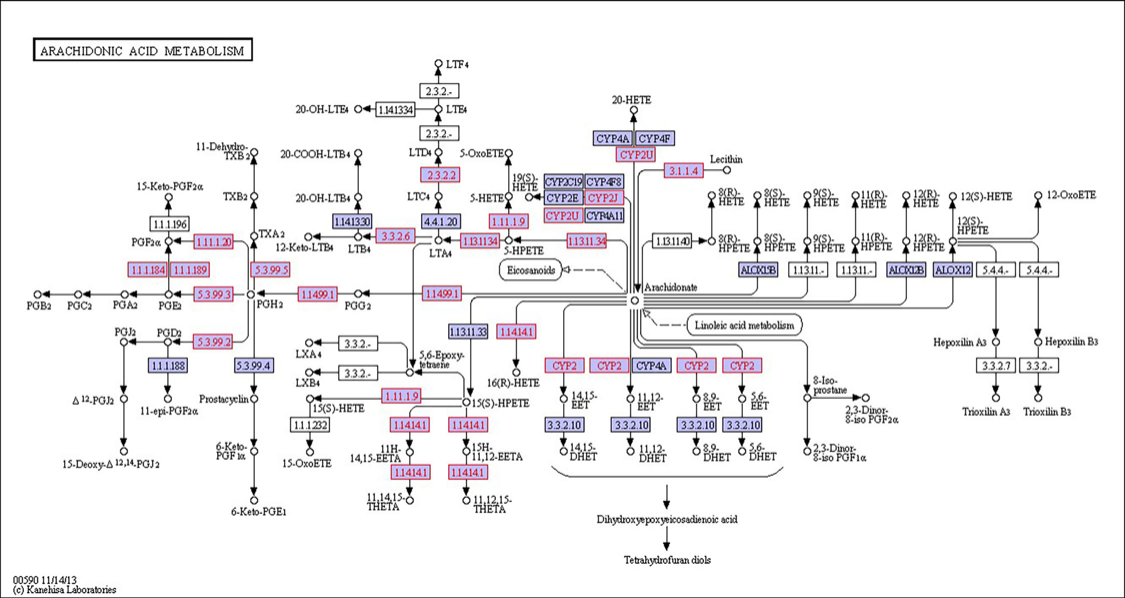
Pathway of arachidonic acid metabolism.

There were 39, 42 and 32 pathways showing significant changes in paired-comparisons of FO vs SBL, BT vs SBL and FO vs BT, respectively and the significant changes of pathways (*P* < 0.05) with the change of gene numbers were showed in S3, S4 and S5 Tables, respectively. The pathways of glycerolipid metabolism (Fig 2), fatty acid biosynthesis (Fig 3), fatty acid elongation (Fig 4), fatty acid degradation (Fig 5), biosynthesis of unsaturated fatty acid (Fig 6), glycerophospholipid metabolism (Fig 7), linoleic acid metabolism (Fig 8) and arachidonic acid metabolism (Fig 9) were changed in all three paired-comparisons, and the above pathways were significantly changed in FO vs SBL and BT vs SBL, but in comparison of BT vs FO, only linoleic acid metabolism was changed significantly. The genes of triacylglycerol lipase, aldehyde reductase, phosphatidate phosphatase, diacylglycerol kinase (ATP) and glycerol kinase were significantly up or down-regulated in the glycerolipid metabolism pathway. In the glycerophospholipid metabolism pathway, many genes were significantly regulated especially in secretory phospholipase A2, phosphoethanolamine N-methyltransferase, lysophospholipase I, glycerol-3-phosphate dehydrogenase (NAD+), lysophosphatidylcholine acyltransferase and ethanolamine kinase. The utilization of polyunsaturated fatty acids was enhanced at low salinity, especially in arachidonate and linoleate. All the significantly changed (*P* < 0.05) KEGG genes were listed in Table 5.

**Table 5:** Summary of the significantly changed gene relevant to lipid metabolism.

gene definition	gene name	gene EC number	FO vs SBL	BT vs SBL	FO vs BT
triacylglycerol lipase	E3.1.1.3	EC:3.1.1.3	+7.53	+6.70	+2.67
fatty acid synthase	FASN	EC:2.3.1.85	not detected	not detected	-1.42
glycerol kinase	glpK, GK	EC:2.7.1.30	+1.17	+1.71	-1.98
glycerol-3-phosphate O-acyltransferase 1/2	GPAT1_2	EC:2.3.1.15	+2.82	+1.14	+2.16
1-acyl-sn-glycerol-3-phosphate acyltransferase	plsC	EC:2.3.1.51	+1.37	+1.95	not detected
phosphatidate phosphatase	PPAP2	EC:3.1.3.4	+6.68	+6.09	-6.19
long-chain acyl-CoA synthetase	ACSL, fadD	EC:6.2.1.3	-1.15	-1.28	-1.08
acyl-CoA oxidase	E1.3.3.6, ACOX1, ACOX3	EC:1.3.3.6	+2.20	+1.21	-2.03
long-chain-acyl-CoA dehydrogenase	ACADL	EC:1.3.8.8	+3.35	+4.02	not detected
palmitoyl-protein thioesterase	PPT	EC:3.1.2.22	+2.44	+1.31	+1.13
acyl-CoA oxidase	E1.3.3.6, ACOX1, ACOX3	EC:1.3.3.6	+1.61	+2.88	-2.03
acyl-coenzyme A thioesterase 1/2/4	ACOT1_2_4	EC:3.1.2.2	-1.40	-2.70	+1.30
glycerol-3-phosphate dehydrogenase (NAD+)	GPD1	EC:1.1.1.8	+2.84	+1.59	+1.25
secretory phospholipase A2	PLA2G, SPLA2	EC:3.1.1.4	+2.61	+3.94	-1.33

## Discussion

In this study, RNA-seq successfully revealed the dietary lipid sources significantly changed the pathways of glycerolipid metabolism, linoleic acid metabolism, arachidonic acid metabolism, glycerophospholipid metabolism, fatty acid biosynthesis, fatty acid elongation, fatty acid degradation and biosynthesis of unsaturated fatty acid. These pathways can be generally categorized into energy metabolism related pathways, cell membrane structure modulation related pathways and other pathways. Since the growth trial was conducted on *L*. *vannamei* at 3 psu, a low salinity causing stress to this marine shrimp, the significant changes of pathways in this study reflect the physiological response of *L*. *vannamei* to different dietary lipid sources in a stressful condition.

### Pathways of lipid metabolism involved into energy supply

**Fig 10:**
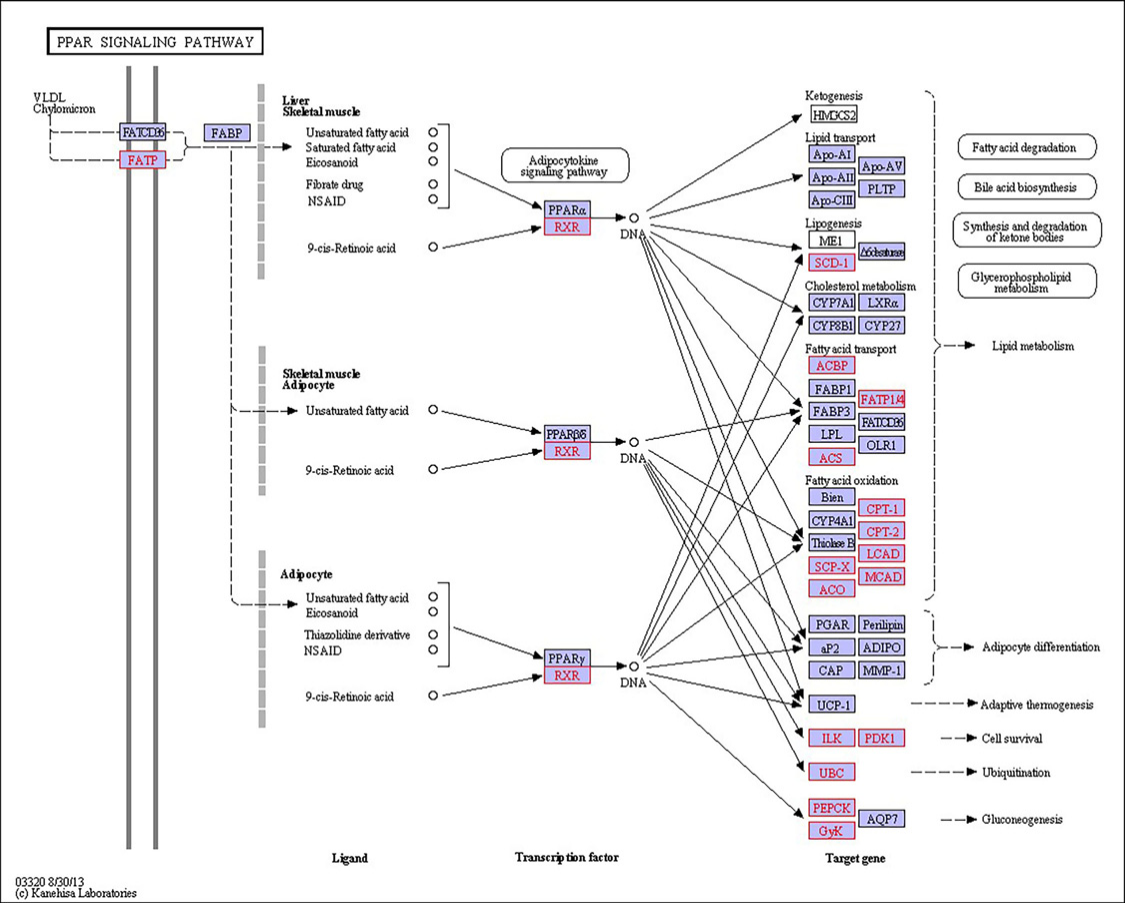
Pathway of PPAR signaling pathway.

Extra energy supply is required in osmoregulation for crustaceans to survive in a habitat with high salinity fluctuation, especially in a low salinity environment [19, 46]. When *L*. *vannamei* are under low salinity stress, the change of osmolality in hemolymph can lead to osmoregulation to counteract salinity shock [47, 48].To keep homeostasis under low salinity, shrimp could obtain extra energy from the diet to maintain osmolality in hemolymph via active ion transport [12, 19, 46], and diet lipid contents have proved of significant roles in this process [20, 35, 49, 50]. In this study, some pathways including glycerolipid metabolism (Fig 2), fatty acid biosynthesis (Fig 3), fatty acid elongation (Fig 4), fatty acid degradation (Fig 5) and PPAR signaling pathways (Fig 10), were mainly involved in energetic adaptation to low salinity stress. It has been proved that crustaceans prefer to use shorter-chain fatty acids to obtain energy through β-oxidation [51] and saturated fatty acid would be primarily used in energy metabolism. In this study, the BT diet contained highest saturated fatty acids with a proportion of 53.86%, and the FO diet (32.66%) had little higher fatty acids than the SBL diet (28.23%). The results of glycerolipid metabolism showed that the gene expression of triacylglycerol lipase was significantly enhanced in all paired comparison of FO vs BT, FO vs SBL and BT vs SBL, and led to fatty acid hydrolyzation from triacylglycerol to fatty acid degradation (Fig 5). Interestingly, the fatty acid synthase gene responsible for synthesis of 18C fatty acid was down-regulated by diet FO compared with diet BT. We speculate that this phenomenon was due to the inhibition effect on the mRNA of fatty acid synthase (FAS) by intake of excess dietary polyunsaturated fatty acids such as DHA and EPA [52, 53], resulting in poorer growth performance of shrimp fed FO than those fed SBL (S6 Table). Meanwhile, glycerol kinase, glycerol-3-phosphate O-acyltransferase, 1-acyl-sn-glycerol-3-phosphate acyltransferase and phosphatidate phosphatase were up-regulated both in the comparisons of FO vs SBL and BT vs SBL, suggesting that the synthesis of triacylglycerol from glycerol was enhanced. In the contrary, the synthesis of triacylglycerol in shrimp fed FO was down-regulated compared to those fed BT. In conclusion, shrimp fed BT had highest capability to synthesize triacylglycerol, followed by those fed FO, and shrimp fed SBL were poorest.

In fatty acid degradation, β-oxidation is the principal pathway to oxidize fatty acid to gain ATP in peroxisomes [54], and all the long-chain acyl-CoA synthetases were down-regulated due to the utilization of fatty acids in β-oxidation. Meanwhile, the acyl-CoA oxidase and long-chain-acyl-CoA dehydrogenase were significantly up-regulated in both FO and BT diet groups compared with the SBL diet group, and the up-regulation in the BT diet group was more than in the FO diet group. Shrimp fed BT had the highest gene expression in saturated fatty acid degradation due to possessing the highest proportion of saturated fatty acid. Fatty acid plays an important role in energy supplementation in osmoregulation [19, 20]. In the fatty acid elongation pathway, the gene expression of palmitoyl-protein thioesterase in shrimp fed FO was up-regulated the most, followed those fed BT and SBL. The palmitoyl-protein thioesterase can remove palmitate groups from cysteine residues in lipid-modified proteins [55] and produce palmitic acid for fatty acid degradation. On the other hand, the peroxisome proliferator-activated receptors (PPARs) are a group of nuclear receptor proteins that function as transcription factors regulating the expression of genes to regulate carbohydrate, lipid, and protein metabolism and especially in fatty acid catabolism [56, 57]. The of gene acyl-CoA oxidase (ACO) higher expressed in shrimp fed FO or BT than those fed SBL, and the expression in the FO group was significantly lower than in BT group (Fig 10). As shrimp in the BT diet group contained the highest saturated fatty acid proportion, saturated fatty acid can be used in producing acyl-CoA to supply energy through oxidation [54]. The high content of saturated fatty acid would promote the production of fatty acid transporters for fatty acid transportation.

Shrimp fed BT possessed the highest saturated fatty acid which could be used as energy supplementation for osmoregulation, but shrimp fed BT showed the poorest growth performance in all three groups. It seems that sole energy intake is not sufficient to overcome the energy loss in osmoregulation. The modification of cell membrane could be another way of adaption for osmoregulation at low salinity.

### Pathways of lipid metabolism related to cell membrane permeability

The structure and permeability of the cell membrane on gills play an important role in osmoregulation for crustaceans to maintain hemolymph osmolality/ion and survive under salinity stress [46, 58, 59]. Previous studies have shown that dietary PUFAs can improve growth and osmoregulation capacity in aquatic animals under osmotic shock [17, 18, 20] because PUFAs are closely associated with cell membrane to increase membrane permeability and enhance fluidity [60, 61]. The modification of fatty acid composition in gills with higher levels of LC-PUFA (usually over 20 carbon atom, especially DHA and EPA) have the potential to increase the gill area and enzymatic efficiency [17, 18] to improve osmoregulatory capacity [62]. Therefore, LC-PUFAs can play a crucial role in osmoregulation, and supplementation of LC-PUFA in the diet should satisfy the need of *L*. *vannamei*. However, we found that shrimp fed FO containing highest LC-PUFAs did not show the best growth performance, but shrimp fed SBL displayed the best growth. Considering the energy supplementation in diets, the BT diet possessed the highest saturated fatty acids (SFAs) but lacked PUFAs. In contrast, the FO diet contained highest PUFAs but lowest SFAs. Shrimp fed either BT or FO diet did not show a satisfactory growth. Although the SBL diet had high SFA and α-linolenic acid (C18:3n-3) to satisfy energy requirement, but this diet was short of LC-PUFA just like the BT diet. Thus, we speculate that the reason why shrimp fed SBL exhibited best growth performance may due to the ability to synthesize LC-PUFAs from α-linolenic acids in *L*. *vannamei*.

Most marine shrimp have a limited ability to synthesize LC-PUFAs [23], but our previously study indicates that *L*. *vannamei* may possess the ability to synthesize DHA and EPA from α-linolenic acids at low salinity [20]. We also found that relevant gene expressions (gene bank accession number: KP271446 and KT305965) in the pathways of fatty acid elongation and unsaturated fatty acids biosynthesis, which supports the above assumption. In the biosynthesis pathway for unsaturated fatty acid pathway, acyl-CoA thioesterase is the crucial gene for synthesizing long-chain unsaturated fatty acid especially DHA and EPA. It is clear that the gene expression in the FO diet group was most down-regulated, followed by the BT diet group, and the SBL diet group was lowest. It is deduced that the shrimp in the SBL diet group need extra long-chain PUFAs and the SBL diet had the highest α-linolenic acids and led to highest gene expression of acyl-CoA thioesterase. However, the FO diet group showed poorest gene expression of acyl-CoA thioesterase because of sufficient long-chain PUFAs in this diet. Therefore, this evidence suggests that *L*. *vannamei* possess the ability to synthesize DHA and EPA fromα-linolenic acids under low salinity stress.

On the other hand, glycerophospholipid is the main component of biological membrane [63], and the glycerophospholipid metabolism pathway significantly changed in this study. The gene expression of glycerol-3-phosphate dehydrogenase was up-regulated most in FO diet group, followed by the BT diet group and the SBL diet group. Glycerone phosphate would increase when the glycerol-3-phosphate dehydrogenase up-regulated, and the glycerone phosphate can be used in glycerolipid metabolism for lipid metabolism to resist osmotic shock [54, 64]. Furthermore, the lecithin was used in linoleic acid metabolism because the gene secretory phospholipase A2 significantly up-regulated, resulting in high production of linoleate/linoleic acid. The linoleate/linoleic acid can be used in arachidonic acid metabolism, and arachidonic acid not only had a positive effect on aquatic animals but also can alleviate osmotic shock [65]. But, the specific mechanism of glycerophospholipid and arachidonic pathway still need further study.

### Other pathways in *L*. *vannamei* under low salinity stress

When shrimp are at low salinity stress, dietary lipids play an important role in osmoregulation [20, 35]. Among three lipid sources in this study, the pathways of osmoregulation differed between the types and contents of fatty acids in the diet. As osmoregulation is a complex process, many pathways are directly or indirectly involved. However, no clear evidence on direct involvement of the pathways were detected during the trial of salinity challenge. Thus, the putative functions of some pathways are briefly discussed.

Amino acids are important osmotic effectors in crustacean [31, 66, 67]. In this study, the pathways of many amino acids were involved such as lysine, valine, leucine and isoleucine. Lysine is metabolized in eukaryotes to yield acetyl-CoA via lysine acetylation [68, 69]. Acetyl-CoA participates in osmoregulation as an intermediate metabolite can indirectly influence ion transfer or energy metabolism and promote “compensatory processes", by producing energy from lipid and carbohydrate metabolism. On the other hand, ketone bodies also play an indispensable role in energy metabolism during the period of low food intake or carbohydrate restriction and energy has to be obtained from breaking down fatty acids in liver [70, 71].

Moreover, steroid hormones are involved in osmoregulation of cetaceans [72] to control metabolism, immune functions and salt and water balance [73–75]. Phosphonates are effective chelating agents that remain stable under harsh conditions, and phosphonates are also regularly used in reverse-osmosis systems [76]. On the other hand, the study of folate on osmoregulation is sometimes seen in plants because plants often face salinity stress in saline soils, and folate is involved in osmoregulation as a metabolite or metabolic intermediate [77, 78]. In this study drug metabolism-cytochrome P450 pathways were found and cytochrome P450 may indirectly influence osmoregulation by regulating arachidonic acid metabolism [79, 80], fatty acid metabolism [81] or other physiological and biochemical processes. However, the interaction or correlation between these pathways in osmoregulation is still not clear and requires further study.

## Conclusion

This study reported the response of *L*. *vannamei* at low salinity to different sources of dietary lipid at the transcriptome level. The transcriptome analysis shows that when *L*. *vannamei* are under osmotic shock, the osmoregulation in shrimp depends on the source and content of fatty acid in the diet. The metabolism of SFA supplies sufficient energy for extra energy demand via β-oxidation of fatty acids. On the other hand, long-chain unsaturated fatty acid will participate in structural change of cell membrane to regulate the permeability and fluidity, and increase the cell membrane area on gills. A series of lipid metabolism pathways have enhanced the capability of *L*. *vannamei* to cope with osmotic shock and have a positive effect on growth and survival. However, osmoregulation is a complex physiological process and involves many pathways. The details of specific lipid metabolism for osmoregulation are still unclear and need further study. Based on the findings in this transcriptomic study, future research should be conducted to understand protein expression, and biochemical and physiological functions in shrimp at low salinity.

## Supporting Information

S1 Fig Length distribution of isogenes by Illumina sequencing. (TIF)

S2 Fig Distribution of isogenes annotated with COG terms. (TIF)

S1 Table Formulation and proximate of experimental diets. (DOCX)

S2 Table Fatty acids composition of experimental diets (% by weight of total fatty acids). (DOCX)

S3 Table The significantly changed KEGG pathway of L. vannamei in FO vs SBL. (DOCX)

S4 Table The significantly changed KEGG pathway of L. vannamei in BT vs SBL. (DOCX)

S5 Table The significantly changed KEGG pathway of L. vannamei in FO vs BT. (DOCX)

S6 Table Growth, survival (%) and body composition (g/kg wet weight) of white shrimp at 3‰ salinities. (DOCX)
