# Supplementary figures and images for: Comparative Transcriptome Analysis in the Hepatopancreas Tissue of Pacific White Shrimp Litopenaeus vannamei Fed Different Lipid Sources at Low Salinity
Source: PLoS One. 2015 Dec 15;10(12):e0144889. doi: 10.1371/journal.pone.0144889 (PMC4686024; doi:10.1371/journal.pone.0144889)

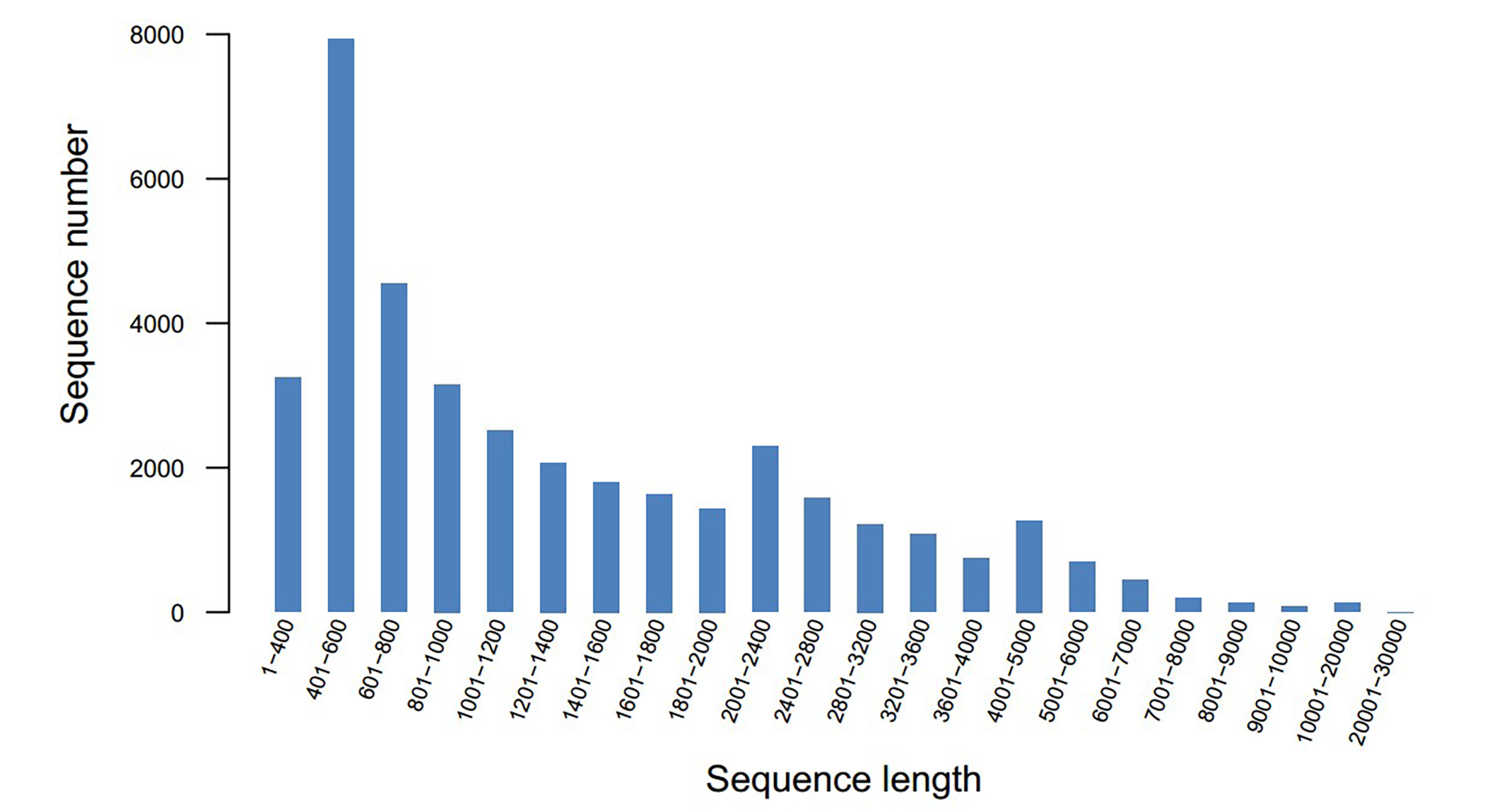

Supplement: S1 Fig — (TIF) [file pone.0144889.s001.tif]

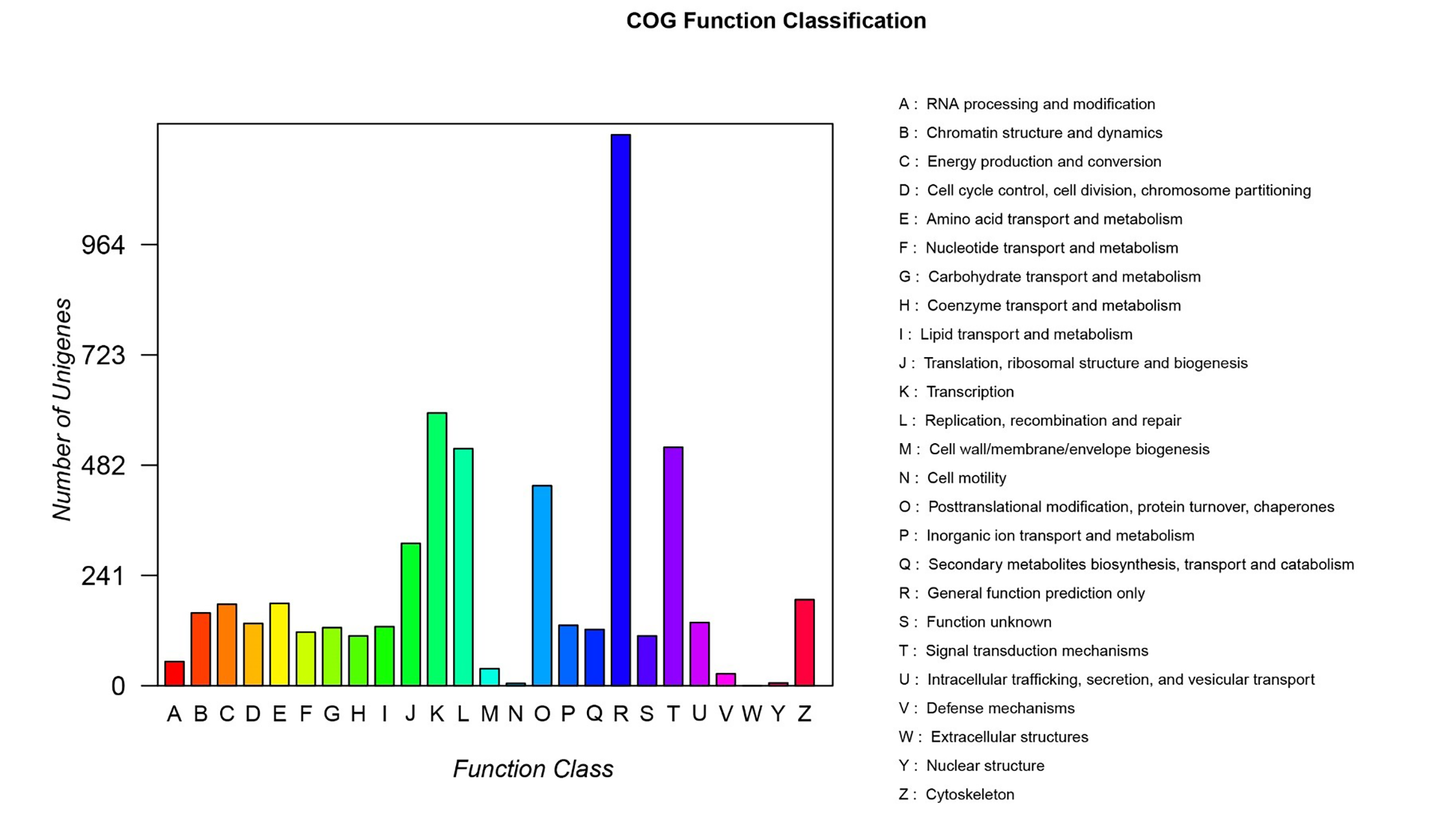

Supplement: S2 Fig — (TIF) [file pone.0144889.s002.tif]
