# Supplementary material for: Comparative Transcriptome Analysis in the Hepatopancreas Tissue of Pacific White Shrimp Litopenaeus vannamei Fed Different Lipid Sources at Low Salinity
Source: PLoS One. 2015 Dec 15;10(12):e0144889. doi: 10.1371/journal.pone.0144889 (PMC4686024; doi:10.1371/journal.pone.0144889)
Supplement: S1 Table — (DOCX) [file pone.0144889.s003.docx]

**S1 Table. Formulation and proximate of experimental diets.**

| Ingredients (g/kg diet) | BT | FO | SBL |
| --- | --- | --- | --- |
| Casein | 320 | 320 | 320 |
| Gelatin | 80 | 80 | 80 |
| Corn starch | 33 | 330 | 330 |
| Vitamin premix2^1^ | 20 | 20 | 20 |
| Mineral premix3^2^ | 5 | 5 | 5 |
| CMC | 30 | 30 | 30 |
| Cholesterol | 5 | 5 | 5 |
| Lecithin | 10 | 10 | 10 |
| Lipid^3^ | 70 | 70 | 70 |
| Vitamin C | 1 | 1 | 1 |
| Amino acid mixture^4^ | 30 | 30 | 30 |
| α-Cellulose | 84 | 84 | 84 |
| Nutrient levels |  |  |  |
| crude protein | 352 | 35.2 | 35.2 |
| crude lipid | 72 | 7.2 | 7.2 |
| gross energy (MJ/kg) | 16.7 | 16.7 | 16.7 |

^1^ Vitamin premix (g/kg premix): thiamin HCl, 0.5; riboflavin, 3.0; DL a-pantothenate, 5.0; nicotinicacid, 5.0; biotin, 0.05; folic acid, 0.18; vitamin B_12_, 0.002; choline chloride, 100.0; inositol, 5.0; menadione, 12.0; vitamin A acetate (20,000 IU/g), 5.0; vitamin D_3_ (400,000 IU/g), 0.002; DL-alpha-tocopheryl acetate (250 IU/g), 8.0; and alpha-cellulose, 866.266.

^2^ Mineral premix (g/100 g premix): sodium dihydrogen phosphate, 21.5; calcium dihydrogen phosphate, 26.5; calcium carbonate, 10.5; Ca-lactate, 16.5; cobalt chloride, 0.001; cupric sulfate pentahydrate, 0.0625; ferrous sulfate, 1.0; magnesium sulfate heptahydrate, 7.0995; manganous sulfate monohydrate, 0.1625; potassium iodide, 0.0167; sodium selenite, 0.0025; zinc sulfate heptahydrate, 3.298.

^3^ lipid:lipid resources including beef tallow (BT), fish oil (FO) and equal combinations of soybean oil +BT+ linseed oil (SBL).

^4^ Amino acid mixture contained the following (g/3 g diet): glycine, 0.6g; l-alanine, 0.6g; l-glutamic acid, 0.6g; and betaine, 1.2g.
