# Supplementary material for: Comparative Transcriptome Analysis in the Hepatopancreas Tissue of Pacific White Shrimp Litopenaeus vannamei Fed Different Lipid Sources at Low Salinity
Source: PLoS One. 2015 Dec 15;10(12):e0144889. doi: 10.1371/journal.pone.0144889 (PMC4686024; doi:10.1371/journal.pone.0144889)
Supplement: S2 Table — (DOCX) [file pone.0144889.s004.docx]

**S2 Table. Fatty acids composition of experimental diets (% by weight of total fatty acids).**

| Fatty acid | BT | FO | SBL |
| --- | --- | --- | --- |
| C16:0 | 23.05 | 21.47 | 13.67 |
| C18:0 | 28.53 | 5.41 | 13.38 |
| ∑SFA | 53.86 | 32.66 | 28.23 |
| C18:1n9 | 34.83 | 23.87 | 24.43 |
| ∑MUFA | 36.93 | 32.49 | 27.30 |
| C18:2n6 | 8.15 | 10.06 | 23.22 |
| C18:3n3 | 0.42 | 3.92 | 20.96 |
| C20:5n3 | 0.12 | 6.63 | 0.10 |
| C22:6n3 | 0.15 | 8.96 | 0.12 |
| ∑PUFA | 9.21 | 34.85 | 44.47 |
| ∑n-3 | 0.69 | 20.77 | 21.17 |
| ∑n-6 | 8.52 | 14.08 | 23.30 |
| ∑n-3/∑n-6 | 0.08 | 1.48 | 0.91 |

Not all analyzed fatty acids fractions were included in this table.

∑SFA: 14:0, 16:0, 18:0, 20:0, 22:0.

∑MUFA: 16:1, 18:1n9, 20:1, 22:1.

∑PUFA: 18:2n6, 18:3n3, 20:2, 20:3n6, 20:4n6, 20:3n3, 20:5n3, 22:2, 22:3, 22:4, 22:5n3, 22:6n3.

∑n-3: 18:3n3, 20:3n3, 20:5n3, 22:5n3, 22:6n3.

∑n-6: 18:2n6, 20:3n6, 20:4n
