# Supplementary material for: Comparative Transcriptome Analysis in the Hepatopancreas Tissue of Pacific White Shrimp Litopenaeus vannamei Fed Different Lipid Sources at Low Salinity
Source: PLoS One. 2015 Dec 15;10(12):e0144889. doi: 10.1371/journal.pone.0144889 (PMC4686024; doi:10.1371/journal.pone.0144889)
Supplement: S3 Table — (DOCX) [file pone.0144889.s005.docx]

| **Pathway name** | **Id** | **Sample number** | **Background number** | **P-Value** |
| --- | --- | --- | --- | --- |
| Drug metabolism - cytochrome P450 | ko00982 | 32 | 63 | 2.05E-12 |
| Metabolism of xenobiotics by cytochrome P450 | ko00980 | 34 | 71 | 3.64E-12 |
| Retinol metabolism | ko00830 | 21 | 45 | 8.65E-08 |
| Folate biosynthesis | ko00790 | 14 | 28 | 4.54E-06 |
| Arginine and proline metabolism | ko00330 | 25 | 75 | 1.09E-05 |
| Drug metabolism - other enzymes | ko00983 | 25 | 76 | 1.41E-05 |
| Pantothenate and CoA biosynthesis | ko00770 | 11 | 22 | 4.91E-05 |
| Glycerolipid metabolism | ko00561 | 20 | 61 | 0.000105 |
| Caffeine metabolism | ko00232 | 10 | 21 | 0.000183 |
| Cysteine and methionine metabolism | ko00270 | 17 | 50 | 0.000207 |
| Glycerophospholipid metabolism | ko00564 | 27 | 98 | 0.000209 |
| Steroid hormone biosynthesis | ko00140 | 12 | 29 | 0.000219 |
| Fructose and mannose metabolism | ko00051 | 19 | 63 | 0.000517 |
| Alanine, aspartate and glutamate metabolism | ko00250 | 15 | 46 | 0.000797 |
| Glutathione metabolism | ko00480 | 20 | 73 | 0.001427 |
| Linoleic acid metabolism | ko00591 | 14 | 44 | 0.001544 |
| Synthesis and degradation of ketone bodies | ko00072 | 11 | 32 | 0.002433 |
| Adherens junction | ko04520 | 31 | 137 | 0.002771 |
| Two-component system | ko02020 | 6 | 12 | 0.002829 |
| Amino sugar and nucleotide sugar metabolism | ko00520 | 24 | 99 | 0.003121 |
| Peroxisome | ko04146 | 28 | 124 | 0.004449 |
| Lysine degradation | ko00310 | 24 | 102 | 0.004694 |
| Insect hormone biosynthesis | ko00981 | 7 | 17 | 0.004842 |
| Aminobenzoate degradation | ko00627 | 9 | 26 | 0.005644 |
| Nicotinate and nicotinamide metabolism | ko00760 | 10 | 31 | 0.006339 |
| Amyotrophic lateral sclerosis (ALS) | ko05014 | 10 | 31 | 0.006339 |
| Renin-angiotensin system | ko04614 | 7 | 18 | 0.007002 |
| Ether lipid metabolism | ko00565 | 11 | 38 | 0.010463 |
| Butanoate metabolism | ko00650 | 13 | 50 | 0.014499 |
| Histidine metabolism | ko00340 | 6 | 16 | 0.015103 |
| Phosphonate and phosphinate metabolism | ko00440 | 4 | 8 | 0.015307 |
| Methane metabolism | ko00680 | 9 | 31 | 0.019383 |
| Lysosome | ko04142 | 40 | 219 | 0.031815 |
| Valine, leucine and isoleucine degradation | ko00280 | 14 | 61 | 0.032748 |
| Valine, leucine and isoleucine biosynthesis | ko00290 | 4 | 10 | 0.036671 |
| Apoptosis | ko04210 | 14 | 63 | 0.042013 |
| Glycine, serine and threonine metabolism | ko00260 | 15 | 69 | 0.043066 |
| Arachidonic acid metabolism | ko00590 | 15 | 70 | 0.048161 |
| Small cell lung cancer | ko05222 | 18 | 88 | 0.049081 |

**S3 Table.** The significantly changed KEGG pathway of *L. vannamei* in FO vs SBL.
