# Supplementary material for: Comparative Transcriptome Analysis in the Hepatopancreas Tissue of Pacific White Shrimp Litopenaeus vannamei Fed Different Lipid Sources at Low Salinity
Source: PLoS One. 2015 Dec 15;10(12):e0144889. doi: 10.1371/journal.pone.0144889 (PMC4686024; doi:10.1371/journal.pone.0144889)
Supplement: S4 Table — (DOCX) [file pone.0144889.s006.docx]

| **Pathway name** | **Id** | **Sample number** | **Background number** | **P-Value** |
| --- | --- | --- | --- | --- |
| Drug metabolism - cytochrome P450 | ko00982 | 28 | 63 | 2.43E-09 |
| Metabolism of xenobiotics by cytochrome P450 | ko00980 | 30 | 71 | 2.89E-09 |
| Linoleic acid metabolism | ko00591 | 18 | 44 | 7.35E-06 |
| Retinol metabolism | ko00830 | 18 | 45 | 1.07E-05 |
| Glycosaminoglycan biosynthesis - keratan sulfate | ko00533 | 10 | 17 | 1.69E-05 |
| Amino sugar and nucleotide sugar metabolism | ko00520 | 28 | 99 | 9.65E-05 |
| Arachidonic acid metabolism | ko00590 | 22 | 70 | 9.73E-05 |
| Drug metabolism - other enzymes | ko00983 | 23 | 76 | 0.00013 |
| Fructose and mannose metabolism | ko00051 | 20 | 63 | 0.000171 |
| Pentose and glucuronate interconversions | ko00040 | 15 | 41 | 0.00019 |
| Serotonergic synapse | ko04726 | 24 | 83 | 0.000205 |
| Renin-angiotensin system | ko04614 | 9 | 18 | 0.000242 |
| Lysine degradation | ko00310 | 27 | 102 | 0.000416 |
| Glutathione metabolism | ko00480 | 21 | 73 | 0.000539 |
| Glycerolipid metabolism | ko00561 | 18 | 61 | 0.000946 |
| Steroid hormone biosynthesis | ko00140 | 11 | 29 | 0.000956 |
| Folate biosynthesis | ko00790 | 10 | 28 | 0.002737 |
| Glycosphingolipid biosynthesis - lacto and neolacto series | ko00601 | 7 | 16 | 0.003207 |
| Caffeine metabolism | ko00232 | 8 | 21 | 0.004586 |
| Lysosome | ko04142 | 44 | 219 | 0.004867 |
| Other types of O-glycan biosynthesis | ko00514 | 12 | 40 | 0.00556 |
| Valine, leucine and isoleucine degradation | ko00280 | 16 | 61 | 0.006454 |
| Synthesis and degradation of ketone bodies | ko00072 | 10 | 32 | 0.008062 |
| Complement and coagulation cascades | ko04610 | 6 | 15 | 0.010601 |
| GABAergic synapse | ko04727 | 17 | 70 | 0.011407 |
| Butanoate metabolism | ko00650 | 13 | 50 | 0.014393 |
| Flavone and flavonol biosynthesis | ko00944 | 6 | 16 | 0.015037 |
| Insect hormone biosynthesis | ko00981 | 6 | 17 | 0.020612 |
| Glycerophospholipid metabolism | ko00564 | 21 | 98 | 0.022057 |
| Riboflavin metabolism | ko00740 | 5 | 13 | 0.023347 |
| Glycosaminoglycan degradation | ko00531 | 12 | 48 | 0.024723 |
| Ascorbate and aldarate metabolism | ko00053 | 7 | 23 | 0.029099 |
| Sphingolipid metabolism | ko00600 | 17 | 78 | 0.031737 |
| Cysteine and methionine metabolism | ko00270 | 12 | 50 | 0.03336 |
| Sulfur metabolism | ko00920 | 6 | 19 | 0.035598 |
| PPAR signaling pathway | ko03320 | 11 | 45 | 0.035923 |
| Vitamin digestion and absorption | ko04977 | 7 | 24 | 0.03639 |
| Valine, leucine and isoleucine biosynthesis | ko00290 | 4 | 10 | 0.036559 |
| Phosphatidylinositol signaling system | ko04070 | 22 | 110 | 0.039843 |
| Starch and sucrose metabolism | ko00500 | 18 | 87 | 0.044154 |
| Porphyrin and chlorophyll metabolism | ko00860 | 13 | 58 | 0.045706 |

**S4 Table.** The significantly changed KEGG pathway of *L. vannamei* in BT vs SBL.
