# Supplementary material for: Comparative Transcriptome Analysis in the Hepatopancreas Tissue of Pacific White Shrimp Litopenaeus vannamei Fed Different Lipid Sources at Low Salinity
Source: PLoS One. 2015 Dec 15;10(12):e0144889. doi: 10.1371/journal.pone.0144889 (PMC4686024; doi:10.1371/journal.pone.0144889)
Supplement: S5 Table — (DOCX) [file pone.0144889.s007.docx]

**S5 Table.** The significantly changed KEGG pathway of *L. vannamei* in FO vs BT.

| **Pathway name** | **Id** | **Sample number** | **Background number** | **P-Value** |
| --- | --- | --- | --- | --- |
| Glycosaminoglycan biosynthesis - keratan sulfate | ko00533 | 7 | 17 | 0.000528 |
| Drug metabolism - cytochrome P450 | ko00982 | 15 | 63 | 0.000578 |
| Aminobenzoate degradation | ko00627 | 8 | 26 | 0.001974 |
| Linoleic acid metabolism | ko00591 | 11 | 44 | 0.002002 |
| Metabolism of xenobiotics by cytochrome P450 | ko00980 | 15 | 71 | 0.002123 |
| Flavone and flavonol biosynthesis | ko00944 | 6 | 16 | 0.002369 |
| Glycosphingolipid biosynthesis - lacto and neolacto series | ko00601 | 6 | 16 | 0.002369 |
| Starch and sucrose metabolism | ko00500 | 17 | 87 | 0.002695 |
| Lysine degradation | ko00310 | 19 | 102 | 0.002777 |
| Pentose and glucuronate interconversions | ko00040 | 10 | 41 | 0.003841 |
| Glycosaminoglycan degradation | ko00531 | 11 | 48 | 0.004167 |
| Lysosome | ko04142 | 33 | 219 | 0.004364 |
| Retinol metabolism | ko00830 | 10 | 45 | 0.007752 |
| Synthesis and degradation of ketone bodies | ko00072 | 8 | 32 | 0.008013 |
| Complement and coagulation cascades | ko04610 | 5 | 15 | 0.009742 |
| Valine, leucine and isoleucine degradation | ko00280 | 12 | 61 | 0.010213 |
| Drug metabolism - other enzymes | ko00983 | 14 | 76 | 0.010424 |
| Various types of N-glycan biosynthesis | ko00513 | 13 | 70 | 0.01244 |
| Folate biosynthesis | ko00790 | 7 | 28 | 0.012855 |
| Apoptosis | ko04210 | 12 | 63 | 0.013155 |
| Pantothenate and CoA biosynthesis | ko00770 | 6 | 22 | 0.013539 |
| Steroid hormone biosynthesis | ko00140 | 7 | 29 | 0.015606 |
| Butanoate metabolism | ko00650 | 10 | 50 | 0.016285 |
| Ascorbate and aldarate metabolism | ko00053 | 6 | 23 | 0.016896 |
| Geraniol degradation | ko00281 | 5 | 18 | 0.021943 |
| Amyotrophic lateral sclerosis (ALS) | ko05014 | 7 | 31 | 0.022312 |
| Transcriptional misregulation in cancer | ko05202 | 15 | 92 | 0.023871 |
| Phosphonate and phosphinate metabolism | ko00440 | 3 | 8 | 0.032224 |
| Small cell lung cancer | ko05222 | 14 | 88 | 0.034271 |
| Cysteine and methionine metabolism | ko00270 | 9 | 50 | 0.041074 |
| Serotonergic synapse | ko04726 | 13 | 83 | 0.045015 |
| Viral myocarditis | ko05416 | 13 | 83 | 0.045015 |
