# Supplementary material for: Comparative Transcriptome Analysis in the Hepatopancreas Tissue of Pacific White Shrimp Litopenaeus vannamei Fed Different Lipid Sources at Low Salinity
Source: PLoS One. 2015 Dec 15;10(12):e0144889. doi: 10.1371/journal.pone.0144889 (PMC4686024; doi:10.1371/journal.pone.0144889)
Supplement: S6 Table — (DOCX) [file pone.0144889.s008.docx]

**S6 Table.** Growth, survival (%) and body composition (g/kg wet weight) of white shrimp at 3‰ salinities.

|  | Survival (%) | Weight gain (%) | Protein (g/kg) | Lipid (g/kg) | Ash (g/kg) | Moisture (g/kg) |
| --- | --- | --- | --- | --- | --- | --- |
| BT | 43.33±4.64^a^ | 335.13±9.80^a^ | 170.1±1.4 | 10.7±1.0 | 25.7±2.4 | 761.8±6.4 |
| FO | 66.96±7.77^bc^ | 343.28±8.06^ab^ | 158.6±8.3 | 13.6±2.2 | 24.2±1.1 | 773.4±11.9 |
| SBL | 86.54±7.54^c^ | 394.36±6.56^c^ | 166.7±2.1 | 14.6±2.0 | 27.6±0.6 | 765.4±3.9 |
| *P* value | 0.012 | 0.011 | 0.559 | 0.063 | 0.499 | 0.707 |

Different letters in the same column represent significant difference (*P* < 0.05).
